# Evolutionary neural architecture search combining multi-branch ConvNet and improved transformer

**DOI:** 10.1038/s41598-023-42931-3

**Published:** 2023-09-22

**Authors:** Yang Xu, Yongjie Ma

**Affiliations:** College of Physics and Electronic Engineering, Northwest Normal University, Lanzhou, 730070 China

**Keywords:** Computer science, Information technology

## Abstract

Deep convolutional neural networks (CNNs) have achieved promising performance in the field of deep learning, but the manual design turns out to be very difficult due to the increasingly complex topologies of CNNs. Recently, neural architecture search (NAS) methods have been proposed to automatically design network architectures, which are superior to handcrafted counterparts. Unfortunately, most current NAS methods suffer from either highly computational complexity of generated architectures or limitations in the flexibility of architecture design. To address above issues, this article proposes an evolutionary neural architecture search (ENAS) method based on improved Transformer and multi-branch ConvNet. The multi-branch block enriches the feature space and enhances the representational capacity of a network by combining paths with different complexities. Since convolution is inherently a local operation, a simple yet powerful “batch-free normalization Transformer Block” (BFNTBlock) is proposed to leverage both local information and long-range feature dependencies. In particular, the design of batch-free normalization (BFN) and batch normalization (BN) mixed in the BFNTBlock blocks the accumulation of estimation shift ascribe to the stack of BN, which has favorable effects for performance improvement. The proposed method achieves remarkable accuracies, 97.24 $\%$ and 80.06 $\%$ on CIFAR10 and CIFAR100, respectively, with high computational efficiency, i.e. only 1.46 and 1.53 GPU days. To validate the universality of our method in application scenarios, the proposed algorithm is verified on two real-world applications, including the GTSRB and NEU-CLS dataset, and achieves a better performance than common methods.

## Introduction

Convolutional neural networks (CNNs), as the foundation of deep learning^1^, have made prominent achievements in various specialized fields, such as image classification^2^, natural language processing^3^ and object detection^4^. The performance of CNNs counts on its architecture to a great extent. However, discovering optimal architectures for different tasks is extremely challenging, because architectures have a large number of parameters and relatively complex structure. At an early stage, the vast majority of the most advanced CNN architectures are hand-crafted by specialists in both neural networks and images processing, such as GoogleNet^5^, ResNet^6^ and DenseNet^7^. Unfortunately, in practice, most users are limited by scarce domain knowledge. Moreover, CNNs are often used to solve specific problems, and once assignments changes, the architecture must be redesigned correspondingly. As a result, neural architecture search (NAS) has attracted unprecedented attention.

NAS aims to automatically design network architectures that achieves the best possible performance with minimal human intervention. Existing NAS algorithms are divided into three main categories: Reinforcement Learning (RL)-based NAS^8^, gradient (GD)-based NAS and Evolutionary Algorithms (EAs)-based NAS^9^. The RL requires large computational resources even on the median-scale dataset like CIFAR10 dataset. Compared with RL, the gradient-based algorithms decrease the consumption of computational resources, but requires to construct a supernet in advance, which have high requirements for expertise. The ENAS searches for superior performance architectures by using EAs. Specifically, EAs are a cluster of algorithms based on biological evolutionary mechanisms, such as natural selection, simulating the evolution of species, to solve optimization problems. Real et al.^10^ conducted a comparison between RL-based NAS and EAs-based NAS, the results demonstrated that EAs-based NAS can converge faster with the same hardware, especially in the initial stage of search.

The methods to design neural network using EAs can be main divided in two branches: Neuroevolution (NE)^11^ and Evolutionary neural architecture search (ENAS). The former exploits EAs to optimize neural networks, and also enables important capabilities, including learning neural network building blocks, the design of topologies, and hyper-parameters^12^. The latter designs neural architectures through Evolutionary Computation (EC) methods. Owing to the evolution of population, the performance of the architecture can constantly improve to a relatively high level on the research task. Recently, ENAS has attracted great attention due to its superior performance. However, since EAs are search methods based on population, plenty of fitness require to be evaluated, leading to ENAS consume enormous computational resources. For instance, CNN-GA^13^ needs to operate on several GPUs for 35 days on CIFAR10 datasets. Therefore, ENAS focus on decreasing the computational resources and accelerating the search process of the neural architecture.

Techniques to reduce computational overheads mainly contain two aspects. One common approach to reduce computational overheads is converting the global search into modular search space^14^. In contrast to the global search space that searches entire neural structure, the modular search space only requires searching a few small cell structures, after which cells are stacked to form the final architecture. Additionally, it is convenient for modular search space to migrate on other tasks, which is usually impossible for global search space. Therefore, compact and flexible is the superiority of the modular search space. The block-based NAS methods generally perform well since they restrict the search space and are inclined to design compact CNN architectures^15^. AE-CNN^16^ constructed a search space based on ResNet block^6^ and DenseNet block^7^ to automatically design CNN architectures. CNN-GA^13^ employed skip connections, enabling deep CNN architectures to be efficiently trained. In the two aforementioned algorithms, an efficient variable-length coding strategy was designed to adaptively search for the unpredictable optimal depth of CNNs. The other method is fitness evaluation. Early versions of NAS^17^ usually find the best neural architecture according to the performance, which is extremely time-consuming since many candidate neural architectures need to be compared. For example, AE-CNN^16^, a block-based search method, takes 27 GPU days on CIFAR10 dataset. Hence, a majority of NAS algorithms focus on how to avoid resource consumption. FP-DARTS^18^ constructs a super network with two-parallel-path to accelerate the training process. EBNAS^19^ proposes an efficient binary neural architecture search algorithm that designs a search space simplification strategy to improve search efficiency.

To search for the network architecture with exceptional performance and further improve search efficiency, this paper proposes an ENAS algorithm combining multi-branch CNNs with improved Transformer. The main contributions of this paper are threefold as follows:

From the perspective of architecture search space, the multi-branch block (MBB) is employed to generate architectures with exceptional performance. MBB extracts abundant features and enhance the representational capacity of a single convolution by combining diverse operations having various receptive fields. Unlike a majority of traditional novel ConvNet architectures, CNN architecture constructed by MBB has a larger model capacity and can be trained to achieve remarkable performance. To search for CNN architecture with the optimal depth, we designed a novel variable-length encoding strategy, which can accommodate various basic units including attention module. Meanwhile, the genetic operators tailored for the novel encoding scheme are designed to avoid the decline of population diversity and improve the optimization efficiency.

A simple yet powerful backbone “batch-free normalization Transformer Block” (BFNTBlock) is proposed to capture long-range dependencies. The proposed BFNTBlock leverages the merits of long range dependence and spatial adaptability derived from MHSA, as well as properties of CNNs, such as inductive bias and local receptive field. In particular, the batch-free normalization (BFN) introduced in BFNTBlock independently normalizes each sample without across batch dimension, which impedes the accumulative estimation shift of BN. This relieves the degeneration of performance if a distribution shift occurs. In addition, an asynchronous computational component is applied to accelerate the design of CNN architectures and discover the optimal network architecture within an acceptable time.

From the results compared with state-of-the-art peer competitors on widely used image classification datasets in NAS: CIFAR10 and CIFAR100, the proposed method is not only computationally efficient but also highly competitive in terms of performance among all compared methods. To further validate the universality in application scenarios, the proposed algorithm is valuated on the GTSRB and the NEU-CLS datasets, and outperforms the commonly used methods.

## Background and related work

In this section, we first briefly describe the classical ENAS algorithms closely related to our work. Also, normalization methods and attention-based architectures are introduced to better understand the proposed algorithm for readers.

### Background

#### Convolution neural networks

Convolution neural network (CNN) is mainly comprised of convolutional layers, pooling layers and fully-connected layers. The parameters of a convolutional layer are the number of feature map, the filter size and the stride size. Specifically, filters, namely convolution kernels, can be viewed as a matrix, are employed to implement convolutional operations on input data. In the process of convolution, the filter horizontally slides with a given stride, multiplying and summing the elements of the filter with the input elements at the corresponding position, then save this result as a value of the output. After that, the filter vertically moves with a given stride until the next horizontal slide. When such operation has performed on the whole image, the output (i.e., feature map) of a convolution layer is obtained.

Pooling layer compresses the feature maps generated by convolution to extract the main features and simplify the computational complexity of the network. According to the return values, pooling operations is divided two types, e.g. maximum pooling and mean pooling. The kernel size, stride size and pooling type are the parameters of a pooling layer. Besides, fully-connected layer is generally the last layer of a CNN to integrate local features.

**Figure 1:**
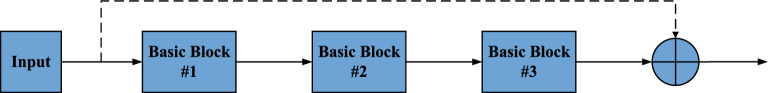
An example of the skip connection.

#### Skip connection

The thought behind the skip connections is to represent the output as a linear superposition of both input and a nonlinear transformation of the input. It refers to those connection of the non-adjacent layers. The skip connections were testified to avoid the Vanishing Gradient (VG) problems^20^ namely the gradient becomes very small or even explodes during back propagation, and train deep neural networks^21^. The promising performance of ResNet^6^ also benefits from the skip connections. As shown in Fig. 1, the dashed line indicates the skip connection, and the symbol $\oplus$ denotes element-wise addition. Combined with the skip connections, deep neural networks (DNNs) can be effectively trained.

### Related work

#### Architecture search space

**Figure 2:**
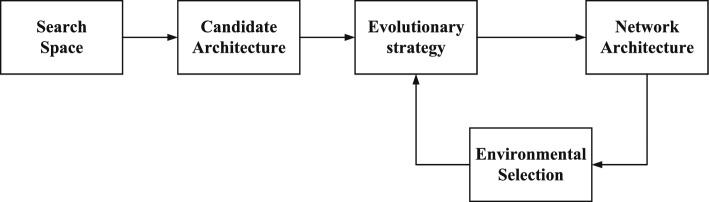
The flowchart of the ENAS.

Generally, given a CNN architecture denoted by by *A* and *a* dataset $D = \left\{ D_{train}, D_{valid}\right\}$, an CNN architecture optimization problem can be formulated as Eq. (1):

1 $$\begin{aligned}&\text {arg min} \text { } L_{valid}(A_{\lambda ,\omega }) \nonumber \\&s.t.\lambda \in \wedge \end{aligned}$$

where $A_{\lambda ,\omega }$ is the architecture with parameters $\lambda$ and weights $\omega$, $L_{valid}(\cdot )$ measures the performance of $A_{\lambda ,\omega }$ on validation data $L_{valid}$. $\lambda$ indicates the definition of an architecture, such as the number of layer; $\omega$ are the weights updated by training the architecture on training data $D_{train}$. $\wedge$ is the set of all possible parameter configurations. Evolutionary neural architecture search (ENAS) is a process of adaptive optimization. Its general framework is shown in Fig. 2. The arrows in Fig. 2 denote the order of execution. EAs have been used for simultaneous optimizing architectures, weights and learning rules of neural networks. For example, William et al.^22^ propose a encoding strategy based on DAG, which is superior to randomly generated CNN architectures. AmoebaNet^23^ uses an modified tournament selection to evolve networks and outperforms hand-crafted models on ImageNet dataset. However, plenty of fitness require to be evaluated in ENAS, which employ enormous computational resources.

The search space defines all possible generated neural architectures, having a vital impact on the performance of architectures and the search efficiency. To an extent, it determines the freedom and the upper-performance limit of neural architecture search (NAS). The search space is determined by the set of predefined operation and the hyper-parameters of the network, such as infrastructure, connection approach, the number of convolutional layer and pooling layer in initial stage. Early NAS approaches^24^ usually utilize the global search space that requires using a search strategy with the ability to search all necessary components of the architecture, which means the optimal neural architecture requires to be discovered within a huge search space. In contrast, modular search space simplifies to only search one or more modules, which can significantly decrease the computational expense. The model generated by means of such modular methods can achieve high performance while also easier to generalize to different tasks.

The modular search space, namely cell or block-based search space has been widely applied to NAS. NASNet^14^ proposes a new search space with excellent transferability, which is composed of normal cells and reduction cells. DPP-Net^25^ utilize a compact search space inspired by mobile CNNs, adopting progressive search to further improve the efficiency. FPNAS^26^ considers the diversity of blocks and demonstrate that stacking different blocks is of great benefit to polish up the performance of neural architecture. Moreover, Hierarchical-Evolution^27^ proposes a scalable of evolutionary search method, which is only need to search for cells with the same structure.

To generate promising architectures and dramatically save computational resources, the multi-branch building block and our proposed BFNTBlock is introduced in search space. There are many advanced architecture designs^5^ have revealed that combining multiple branches with different complexities can enrich the feature space and improve performance. The multi-branch block applied in the proposed algorithm possess various branches with different representational capacity, which is beneficial to extract abundant abstract features, thereby generating highly competitive structures. The architecture search space in this paper uses the chain-structured space based on building blocks (CNN architecture are constructed by these blocks in sequence), to achieve an optimal search for the construction sequence and the depth of network.

#### Normalization methods

As normalization plays a vital role in the comprehensive performance of the network, increasing researchers concentrates on building a normalization module. Batch normalization (BN)^28^ uses mini-batch statistics to normalize the activation during training, but employs the estimated population statistics in the process of inference. It has been broadly applied in architectures^29,30^ while successfully generalize throughout various areas^31^. However, it suffers from problems when solving certain tasks^32^. One limitation is its small-batch-size problem - the error of BN rapidly grows as batch size constantly dcreases^33^. Besides, the performance of batch network significantly degrades if there is a covariate shift between the training and test data^34^.

As such, various batch-free normalization (BFN) have been proposed to address above issues^35,36^, which perform the same normalization operation for each sample during the process of training and inference. In addition, combining estimated population statistics with mini-batch statistics is an effective approach to normalize during training^37,38^. These works probably outperform BN within a small batch^39^, but typically have weak performance within moderate batch. Several works have proposed to construct a normalization module with different normalization methods in a layer. Switchable normalization (SN)^39^ switches among various normalization methods according to their importance weights. By introducing the sparsity constraints^40^ and whitening operation^41^, this idea is further extended.

In contrast to these methods aiming to design a normalization module in a layer, our proposed BFNTBlock constructs BN and BFN in different layers. It is motivated by that a BFN can avoid the degeneration of test performance by relieving the estimation shift of BN. Note that the estimation shift of BN is defined as the discrepancy between the estimated population statistics and the expected statistics^42^.

#### Attention-based architectures for vision

CNNs have obtained significant achievements in computer vision area. A plenty of spatial and channel-wise features can be extracted by the convolution operation in CNNs. In order to extract important features while neglecting useless or conflict with important features, an increasing number of researchers concentrate on attention mechanism. It can allocate the available resources to selectively process notable features and upgrade the performance of CNNs.

The pioneering SENet^43^ is the milestone of attention mechanism area. The method proposed a lighter attention module, enabling networks to concentrates on important channels, which significantly improved the performance of many CNNs with little computing expense. CBAM^44^ infers attention maps along mutually independent channel and spatial dimensions. DA-Net^45^ adaptively integrated local features with global dependencies by combining two different attention modules. GSoPNet^46^ proposes the global second-order pooling to enhance non-linear capability of CNNs. ECA-Net Attention^47^ proposes local cross-channel interaction and utilizes 1D convolution to generate attention. Furthermore, self-attention, first introduced in natural language processing^48^, has shown great competitiveness in the fields of image generation^49^.

Above attention mechanisms provide many novel approaches to generate channel or spatial attention. However, the small-batch-size problem and the estimation shift of BN in a batch normalized network are ignored. Consequently, we shift the study focus from accuracy improvement to robustness. To achieve this, we introduce the BFN to restrain the accumulation of estimation shift caused by the stack of BN. In particular, such design of BFN and BN mixed in BFNTBlock avoid the above the above mentioned drawbacks of BN, enabling network architectures to stably operate within a wide range of batch sizes.

#### Multi-branch Architectures

Inception^29^ adopts a multi-branch structure to enrich the feature space, demonstrating various receptive fields and the combination of diverse branches have a positive impact on performance improvement. Inspired by the idea of multi-branch topology, the multi-branch block (MBB) is introduced, and the merits lies in: (1) MBB can be deemed as a plug-and-play module, effortlessly being intergrated into various convolution-based architectures. (2) each branch of MBB can be converted into a convolutional layer (denoted as conv), so that MBB can be combined into a conv, which is more efficient than common Inception architecture.

#### Transformer-based vision models

Transformer was proposed in natural language processing (NLP)^3^, which bridge the deficiencies of CNNs in capturing global information. The pioneering work ViT^50^ divides an image into non-overlapping patches, revealing that Transformer models could obtain SOTA image recognition performance on large-scale datasets. However, when the training data is insufficient, ViT performs unfavorably owing to the lack of inductive bias. To address this issue, CvT^51^ introduces convolution to incorporate the translation invariance and inductive bias properties into ViT architecture. To leverage the desired properties in CNNs and avoid the small-batch-size problem of batch normalization (BN), a novel “batch-free normalization Transformer Block” (BFNTBlock) is designed to achieve better generalization and capacity.

## The proposed algorithm

In this section, the framework of proposed algorithm is firstly presented, then detail its main components and design the corresponding network architecture for image classification tasks.

### Algorithm overview


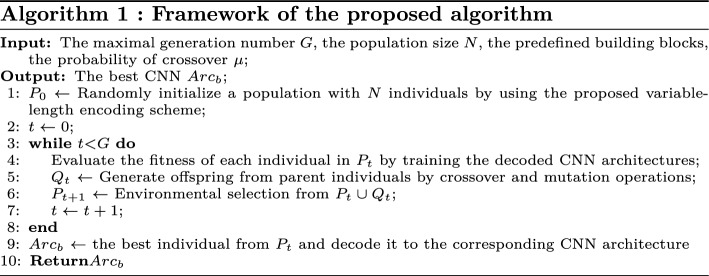


Algorithm 1 presents the framework of proposed algorithm. During the process of evolution, a population with N individuals is randomly initialized and use genetic coding strategy to encode each individual in population (line 1). Then, a counter for current generation is initialized to zero (line 2). Next, the fitness of each individual is evaluated (line 4). After that, the parent individuals are chosen according to fitness, while a new offspring population is produced by the crossover and mutation operators (line 5). Then, individuals in the next generation are opted from current population by environmental selection (line 6). At length, the counter is increased by one, and the evolution continues until the counter exceeds the maximal generation. The optimal individual in the last generation population is decoded to corresponding network and sufficiently trained to obtain the final CNN architecture.

**Figure 3:**
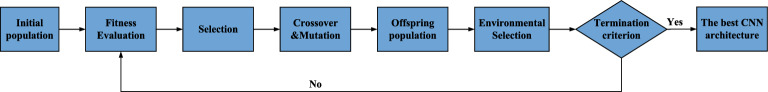
The framework of the proposed algorithm. The individual in population is constructed on multi-branch block and Batch-free Normalization Transformer Block. Evolutionary algorithm is used to search for the optimal CNN architecture. In fitness evaluation, individual performance is predicted on an asynchronous computational component.

As shown in Fig. 3, the workflow of proposed algorithm follows the standard genetic algorithm (GA). Note that GAs with inherent biological mechanism only provide a unified framework to solve optimization problems. Hence, the components regarding biological mechanism require to be specifically designed when GAs are applied on particular tasks. We design a variable-length encoding strategy and genetic operators (containing crossover and mutation) to achieve efficient search.

### Search space

A CNN architecture generally contains multiple hyper-parameters, such as the number, types, and connections of layers in an architecture; the stride, kernel size and number of filters of each layer. In this paper, a novel modular search space based on the MBB and BFNTBlock is designed to achieve better generalization and capacity. The details of MBB and BFNTBlock are shown in Sects. "Multi-branch block" and "Batch-free normalization transformer block", respectively.

#### Multi-branch block

Multi-branch block (MBB) is a robust building block with a multi-path topology. It enriches the feature space by combining different branches of different scales and complexities, including multi-scale convolution, mean pooling and branch addition, thus enhancing the representation capability of a network model. MBB indicates that average pooling and various convolution are more effective, as they provide paths with different complexities, and enable more training-time nonlinearity.

Compared with the classical CNNs such as ResNet^6^ and DenseNet^7^, various paths with different complexities in the DBB expand the receptive field, CNN architecture built on MBB has a more abundant feature space and larger model capacity. Furthermore, some regular convolutional layers can be replaced with DBB to build more complicated micro-structures with no extra inference-time costs.

#### Batch-free normalization transformer block

**Figure 4:**
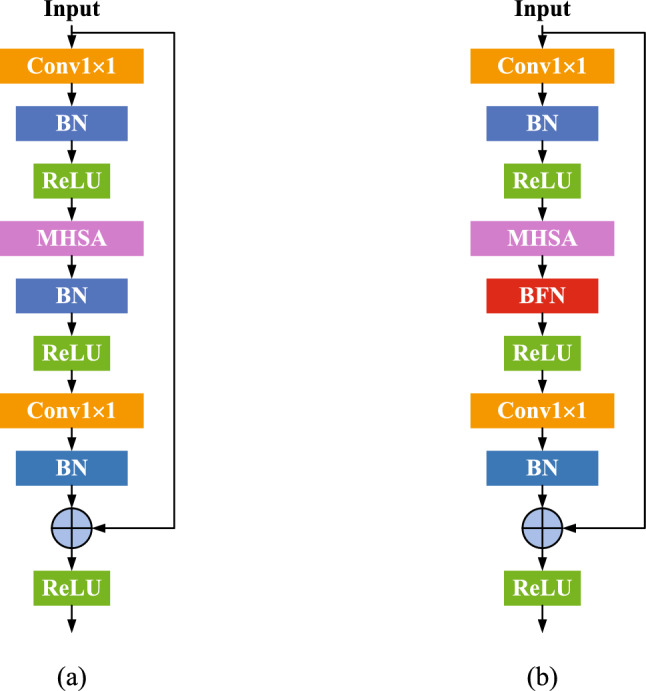
Original Bottleneck (**a**) vs our ’BFNTBlock-P2’ (**b**) that replaces the second BN of a bottleneck with a BFN.

Batch normalization (BN) perform standardization within mini-batches, while improves the conditions of optimization and accelerates training^52^. However, small-batch-size problem and estimation shift are notorious drawbacks of it, which has detriment effects on test performance. To avoid the above demerits of BN and enable network architectures to have strong robustness, we introduce batch-free normalization (BFN) into the BoTNet^53^. Since the BoTNet is a robust backbone, by substituting the convolutions in a ResNet block with global self-attention, the performance of baseline is markedly improved.

The design of BFNTBlock is illustrated as follows: (1) Employ convolutions to efficiently extract abstract features from images; (2) Use self-attention, i.e. MHSA to process and aggregate the features captured by convolutions, while capture global information. (3) Use BFN to avoid the small-batch-size problem of BN. Such a hybrid design can extract local and global features, while enables a network to stably operate within a wide range of batch size. Fig. 4b displays the proposed ’BFNTBlock-P2’ in which we substitute the second BN layer with BFN in the bottleneck (Fig. 4a). Considering the performance of group normalization (GN)^36^ is stable within a wide range of batch size while independently computes the mean and variance for each group, BFNTBlock employs GN as BFN (denoted as BFNTBlockGN). The proposed BFNTBlock with BN and BFN mixed is able to block the accumulation of estimation shift caused by BN. There are a few differences between the proposed BFNTBlock and ordinary Transformer: (1) Normalization: Transformers use Layer Normalization^54^ while BFNTBlock employs BN^28^; (2) Non-Linearities: Transformers possess one non-linearity in the Feed Forward Networks (FFN) block, while the BFNTBlock with ResNet structure enables to use three non-linearities.

#### Population initialization

Population initialization is a prerequisite for the subsequent process of evolution. In this paper, the CNN architectures are randomly initialized and constructed based on multi-branch block, BFNTBlock, pooling block and fully connected layer. The muti-branch block is composed of two hybrid layers, each of which possess various paths with different complexities and scales. These modules are combined in a sequential fashion to form final architectures.

Encoding enables EAs with the ability to model and solve problems. In the proposed algorithm, for muti-branch block, the filter and the stride size of convolutional layer are set to $3 \times 3$ and $1 \times 1$ based on the configuration of the classic CNNs^6^. For pooling layers, a random number within the range of [0, 0.5) represents max pooling, while in range of [0.5, 1) is mean pooling. The kernel and stride size of pooling layers are 2, which means halves the input dimension every time. The type of multi-branch block and BFNTBlock are set to 1. For pooling block, merely set its type to 2. Considering deep layers possess abundant semantic information^55^ and powerful positional information^56^, we insert the proposed BFNTBlock at the tail of individual.

**Figure 5:**
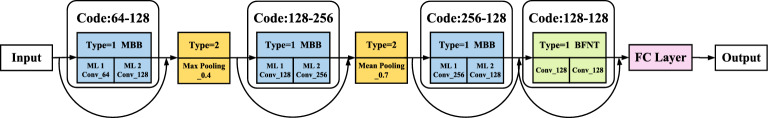
An example of encoding a CNN architecture. The numbers above each layer are the codes of the corresponding layer. The entire encoding of a CNN consists of the codes representing each layer.

An example of encoding a CNN architecture is shown in Fig. 5, which contains three multi-branch blocks (denoted as MBB), namely it has six mixed layers (mixed layer denoted as ML, and 1, 2 indicates number), one BFNTBlock (denoted as BFNT), two pooling blocks and one fully connected layer. The entire code is “64-128-0.4-128-256-0.7-256-128-128-128”, representing a CNN with a depth of 10.

### Search strategy

#### Fitness evaluation


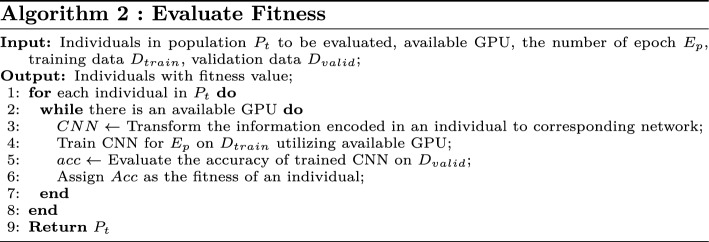


The fitness of individuals, calculated based on the encoded information in individuals, is a quantitative measurement to indicate how well they accommodate to the environment. In the proposed algorithm, we treat the classification accuracy on validation dataset after training as the individual fitness. Algorithm 2 details the fitness evaluation of the proposed algorithm. First, each individual is decoded to corresponding CNN architecture (line 3). After that, the CNN architecture is trained on the training set using available GPU (line 4). Finally, the trained CNN is evaluated on the validation data (line 5), and the obtained classification accuracy is the fitness (line 6).

Since the proposed algorithm is an population-based methods while training CNN architectures is extremely time-consuming, an asynchronous computational component, namely a GPU-based parallel computation platform, is introduced to accelerate the fitness evaluation and make full use of computational resource. By executing sub-problems on different computational platforms in parallel, the total processing time is remarkably reduced. As the fitness evaluation is independent, exactly satisfying the service condition of this technique, such an computational component is introduced. This implies the fitness of multiple individuals can be simultaneously evaluated on different available GPUs, so that the total time of fitness evaluation is dramatically decreased.

#### Evolutionary operators

**Figure 6:**
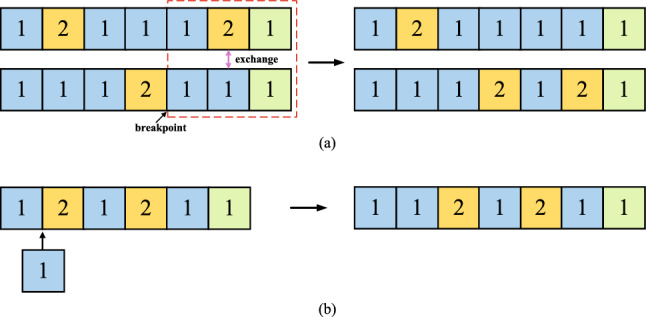
(**a**) Crossover operation. (**b**) Example of the “adding a multi-branch block” mutation. Boxes denote basic building blocks.

The evolutionary operators (include crossover, variation and selection) directly determine the generation of offspring. The crossover and mutation operators are used to search the gene space, while the selection operator is used to select better individuals and guide the search direction. An example of crossover operation is shown in Fig. 6a, each parent individual is separated into two components at the random position (denoted as red arrow), then two parts from different individuals are exchanged to produce offsprings. The mutation operation conducts on one offspring individual with a predefined probability. The mutation types in the proposed algorithms are as follows: adding or removing a block, modifying the parameter values of a block, changing the type of pooling. The “adding a block” mutation is defined with a higher probability while the equal probabilities are used for other mutation operations.

An example of “adding a block” mutation is presented in Fig. 6b. Note that the optimal depth of an architecture is achieved by mutation operators. The crossover operator with global search capability is the primary operator while the mutation operator is the auxiliary operator due to its local search capability. Through the complementation of both operators, our algorithm has a balanced global and local search capability, which assists the proposed algorithm to discover the optimal architecture. Note that the optimal depth of an architecture is achieved by mutation operators. In the proposed algorithm, the optimal depth can be discovered through the “adding or removing a block” operation, which increases or decreases the depth of the CNN.

During the process of environmental selection, a population with N individuals is selected from the current population based on the fitness, serving as parent individuals for the subsequent evolution. To enable population with both convergence and diversity, the binary tournament selection is used to purposely chosen individuals, which their fitness has remarkable differences. Meanwhile, according to the elitism strategy in EAs^57^, the best individual is added into next population to prevent the degradation of the population as the evolution proceeds.

## Experiment design

### Peer competitors

To validate the performance and efficiency of the proposed algorithm, various state-of-the-art NAS algorithms are selected for comparison. According to search strategies, the peer competitors are divided into three categories: manually-designed methods, non-EAs-based methods and EA-based methods.

The first refers to the state-of-the-art manually designed CNNs, including ResNet-101^6^, Wide-ResNet^58^, Pre-ResNet^59^, DenseNet^7^, ResNext-29^60^, SENet^43^, ShuffleNet^61^, MobileNetV2^62^ and IGCV3-D^63^.

The second covers non-EAs-based NAS algorithms. Specifically, the compared algorithms include NAS V3^8^, SNAS^64^, NASNet^14^, ENAS^65^, MetaQNN^66^, Proxyless-NAS^67^, DARTS^68^, RC-DARTS-C42^69^, Block-QNN-S^70^, FPNAS + Cutout^26^, Path-level EAS^71^, PNAS^72^, RENAS^73^, AdaptNAS-S^74^, Firefly^75^, BANANAS^76^, NAGO^77^ and GP-NAS^78^.

The third are ENAS algorithms, including Large-scale Evo^24^, Hierarchical Evolution^27^, AE-CNN^16^, CNN-GA^13^, AE-CNN+E2EPP^79^, NSGANet^80^, AmoebaNet-A^23^ LEMONADE^81^, SI-EvoNet^82^, FPSO^83^ and EffPNet^84^.

### Benchmark datasets

CNNs are commonly performed image classification tasks. For CNN architecture design algorithms, the most used image classification benchmark data sets are CIFAR10 and CIFAR100^85^. Meanwhile, the performance on both datasets are usually used as a criterion to measure deep learning algorithms. Hence, CIFAR10 and CIFAR100 are selected as benchmark data sets.

CIFAR10 and CIFAR100 consists of natural objects. Both are widely applied in image classification tacks. The training set and test set have 50,000 images and 10,000 images respectively, and each category has an equal number of images. The disparity between them is that CIFAR10 covers 10 categories, while CIFAR100 comprises 100 classes.

### Parameters settings

For the proposed algorithm, the parameters follow conventions to ensure applicability. Specifically, the probabilities of crossover and mutation are set to 0.9 and 0.2 respectively, as suggested in^9^. During training phrase, the routine in^6^ is employed, i.e. the individual is trained for 350 epochs by the SGD with the learning rate of 0.1 (divided by 10 at the 149-th, 249-th epoch) and the momentum of 0.9. Indeed, such settings are employed by comparison algorithms.

The available number of feature maps is set to $\{64, 128, 256\}$, as designed in the state-of-the-art CNNs. The population size and the number of evolutional generation are 20 and 10 respectively. For three available mutation operations, the probability of “adding a block” mutation is set to a higher probability, while other mutation operations have the equal probabilities. Theoretically, a deeper CNN architecture may hold a more powerful capability. All experiments are performed on two Nvidia GeForce RTX 2080Ti GPU cards, and the PyTorch 1.8 is selected as the deep learning framework. The proposed algorithm refers to the open source code supplied by Y. Sun^13^.

## Results and discussion

In this section, we firstly present the overall comparison results between the proposed algorithm and peer competitors, while the evolutionary trajectories are displayed to demonstrate the convergence of the proposed algorithm within the parameter settings. Then, the discovered optimal CNN architectures on CIFAR10 and CIFAR100 datasets are shown. Again, we conduct experiments on various attention mechanisms to certify the superior performance and adaptability of proposed BFNTBlock. To validate the effectiveness of our algorithm in real applications, we perform experiments on two application scenarios. Finally, ablation studies are conducted to verify the contribution of each component in our algorithm for performance improvement.

### Overall results

We investigate our method and the compared algorithms in terms of test classification accuracy, the parameters and FLOPs (if available) of output architecture and consumed computational cost. In particular, referring to existing studies, the “GPU Days”^86^ is selected as the quantitative metric for computational cost. The comparison results grouped into three different categories are displayed in Table 1. The symbol “-” implies that the corresponding algorithm does not publicly report results. “SMBO” stands for the “sequential model based global optimization”. “BO” represents the “Bayesian optimization”. Note that the results of competitors are extracted from their seminal papers, since the results in papers are usually the best.

As observed in Table 1, the proposed algorithm outperforms all state-of-the-art handcrafted CNNs on CIFAR10. These methods use well-designed building blocks to improve the compactness and the performance of the architecture, such as Dense block in DenseNet and MBConv block in MobileNetV2. It is shown that the proposed algorithm obtains $3.67\%$ and $0.7\%$ accuracy improvements over ResNet-101 and DenseNet on CIFAR10 dataset, respectively, while having far fewer parameters than DenseNet (4.73M < 25.6M). Moreover, the FLOPs of the proposed algorithm is much smaller than that of the Wide-ResNet and DenseNet. Compared with ResNext-29, our method shows better performance, and has a smaller parameters. In addition, our proposed algorithm achieves the highest classification accuracy among Wide-ResNet, Pre-ResNet, SENet, MobileNetV2, ShuffleNet, IGCV3-D on CIFAR10. On CIFAR100, the proposed algorithm employs $84\%$, $49\%$ and $45\%$ fewer parameters compared to Wide-ResNet, SENet and Pre-ResNet, respectively, and improves performance by $0.56\%$, $2.77\%$ and $2.77\%$, respectively. Though the performance of our algorithm is inferior to those of DenseNet and ResNext-29, the number of parameters is dramatically decreased. The proposed algorithm also outperforms ResNet-101, MobileNetV2, ShuffleNet, IGCV3-D in terms of the classification accuracy. Compared to the lightweight networks, MobileNetV2 and ShuffleNet, the accuracy of our algorithm achieve significant improvements on both dataset.

Compared with non-evolutionary NAS methods in the second category, the proposed algorithm consumes fewer GPU days than all RL-based NAS algorithms except ENAS and FPNAS + Cutout, which uses a sharing parameters approach to accelerate convergence. Moreover, it is observed that, our proposed algorithm outperforms NAS V3 ($1.71\%$), ENAS ($0.18\%$), MetaQNN ($4.16\%$), NASNet-B ($0.97\%$), Block-QNN-S ($1.62\%$), RENAS ($0.12\%$), Path-level EAS ($0.23\%$), FPNAS + Cutout ($0.25\%$) and PNAS ($0.65\%$) on CIFAR10 datasets, while merely consumes about one GPU days. Compared with GD-based algorithms, i.e. SNAS, DARTS, RC-DARTS-C42, Firefly and AdaptNAS-S, our method achieves competitive performance on CIFAR10. However, GD-based methods propose a method to enable the search space continuous in order to optimize the architecture using SGD, which requires a huge hand-crafted CNN as the building block. Once hand-crafted CNN is smaller than the optimal one, GD-based methods will never discover the best CNN architecture. There are no such limitations for the proposed algorithm, which designs promising CNN architectures without domain knowledge. Though our proposed algorithm obtained a slightly worse classification than Proxyless ($-0.68\,\%$), the proposed algorithm has fewer parameters (4.7M < 5.7M) and much less GPU days. For BO-based NAS algorithm, the proposed algorithm surpasses NAGO, GP-NAS in accuracy, and obtains competitive performance with BANANAS in much fewer computational cost. On CIFAR100, our method achieves better performance compared to MetaQNN ($7.2\%$), Block-QNN-S ($0.71\%$), SNAS ($0.15\%$) and NAGO ($0.76\%$). Though the classification accuracy of the proposed algorithm is bit lower than DARTS, but the search only takes 1.53 GPU days. In the second category, the proposed algorithm have the lowest number of FLOPs.

Compared to the proposed algorithm, the major limitation of the non-evolutionary NAS algorithms is the extensively requirement of domain expertise for users. For instance, the CNNs generated by Block-QNN-S cannot be directly used, which must be inserted into a larger hand-crafted CNN in advance, and the final performance of Block-QNN-S depends on whether the larger network is properly designed. The reasons for the proposed algorithm outperforming NAS V3 and Meta-QNN can be summarized as follows. First, there are not apply the crossover operator in Large-scale Evolution, losing the local search ability. In addition, because NAS and Meta-QNN are designed based on RL, lacking the process of fitness evaluation, which often consume more computational resources.

**Table 1:** Comparison of the proposed algorithm with peer competitors in terms of accuracy ($\%$), the number of parameters(M), and the consumed GPU days on CIFAR10 and CIFAR100 datasets.

Architecture	CIFAR10		CIFAR100		Parameters	GPU days	Search method
	ACC	FLOPs	ACC	FLOPs			
ResNet-101	93.57	–	74.84	–	1.7	–	Manual
Wide-ResNet	95.83	–	79.50	5248	36.5	–	Manual
Pre-ResNet	95.36	–	77.29	–	10.3	–	Manual
DenseNet	96.54	9388	82.82	–	25.6	–	Manual
ResNext-29	96.42	–	82.69	–	68.1	–	Manual
SENet	95.95	–	77.29	–	11.2	–	Manual
MobileNetV2	94.56	–	77.09	–	2.1	–	Manual
ShuffleNet	90.87	–	77.14	–	1.06	–	Manual
IGCV3-D	94.96	–	77.95	–	2.2	–	Manual
NAS V3	95.53	–	–	–	7.1	22,400	RL
ENAS	97.06	–	–	–	4.2	0.5	RL
MetaQNN	93.08	–	72.86	–	–	100	RL
Block-QNN-S	95.62	–	79.35	–	6.1	90	RL
Proxyless NAS	97.92	–	–	–	5.7	1,500	RL
NASNet-B	96.27	–	–	–	2.6	2,000	RL
FPNAS + Cutout	96.99	–	–	–	5.8	0.83	RL
Path-level EAS	97.01	–	–	–	5.7	200	RL
RENAS	97.12	–	–	–	3.5	6	RL+EA
SNAS	97.15	–	–	–	2.8	1.5	GD
SNAS	–	–	79.91	422	2.8	1.5	GD
DARTS	97.00	547	–	–	3.3	1.5	GD
DARTS	–	–	82.46	528	3.4	4	GD
RC-DARTS-C42	97.19	–	–	–	3.3	1	GD
Firefly	97.27	–	–	–	3.3	1.5	GD
AdaptNAS-S	97.50	–	–	–	5.3	2	GD
PNAS	96.59	–	–	–	3.2	150	SMBO
BANANAS	97.36	–	–	–	–	11.8	BO
NAGO	96.60	–	79.30	–	–	–	BO
GP-NAS	96.21	–	–	–	–	0.9	BO
Large-scale evolution	94.60	–	–	–	5.4	2,750	EA
Large-scale evolution	–	–	77.00	–	40.4	2,750	EA
LEMONADE	97.42	–	–	–	13.1	90	EA
AmoebaNet-A	96.66	–	81.07	–	3.3	3,150	EA
AE-CNN+E2EPP	94.70	–	–	–	4.3	7	EA
AE-CNN+E2EPP	–	–	77.98	–	20.9	10	EA
NSGANet	97.25	1290	–	–	3.3	4	EA
NSGANet	–	–	79.26	1290	3.3	8	EA
Hierarchical evolution	96.37	–	–	–	15.7	300	EA
CNN-GA	96.78	-	-	-	2.9	35	EA
CNN-GA	–	–	79.47	–	4.1	40	EA
AE-CNN	95.30	–	–	–	2.0	27	EA
AE-CNN	–	–	77.60	–	5.4	36	EA
SI-EvoNet	96.02	–	–	–	0.51	0.46	EA
SI-EvoNet	–	–	79.16	–	0.99	0.81	EA
FPSO	95.16	–	–	–	0.7	1.25	EA
EffPNet	96.51	–	81.51	–	2.54	$<3$	EA
**Proposed**	**97.24**	**167**	–	–	**4.73**	**1.46**	EA
**Proposed**	–	–	**80.06**	**260**	**5.71**	**1.53**	EA

Bold highlights the performance of our algorithm.

For ENAS algorithms in the third category, the proposed algorithm shows superior performance over Large-scale Evolution, AmoebaNet-A, AECNN+E2EPP, Hierarchical Evolution, CNN-GA, AE-CNN and FPSO on CIFAR10, but slightly worse than LEMONADE ($-0.18\%$). Note that Large-scale Evolution and Hierarchical Evolution accelerate search phrase by hardware distributed computation, while CNN-GA and AE-CNN design search space based on efficient building blocks to decrease computational cost. However, they still consumes enormous computational resources. Compared to these ENAS algorithms with high computational costs, the proposed algorithm only takes 1.46 and 1.53 GPU days on CIFAR10 and CIFAR100, respectively. In addition, AE-CNN+E2EPP uses an random forest-based performance predictor to accelerate the process of fitness evaluation, but the proposed algorithm still consumes considerably less GPU days than AE-CNN+E2EPP without using any training tricks, which indicates CNN architectures searched by proposed algorithm are well compact. Compared with the lightweight architecture SI-EvoNet, the performance of proposed algorithm is significantly improved ($1.22\%$). In particular, the proposed algorithm with fewer GPU days achieves competitive classification accuracy compared to the classical NSGANet. For EffPNet, the proposed algorithm is substantially lower in search cost, and achieves better performance on CIFAR10. On CIFAR100, our method perform much better than Large-scale Evolution and AE-CNN+E2EPP, while only has 5.71M parameters, saving $86\%$ parameters compared to Large-scale evolution. Moreover, the proposed algorithm outperforms NSGANet, CNN-GA, AE-CNN and SI-EvoNet. Compared with AmoebaNet-A and EffPNet, the performance on CIFAR100 is weaker against it, but our method consumes much less GPU Days.

In summary, our proposed algorithm outperforms the advanced manually designed CNNs and most of ENAS algorithms in terms of classification accuracy, the number of parameters and the computational overheads. Meanwhile, the discovered architecture is more lightweight and compact than most of the state-of-the-art CNNs. Moreover, the proposed algorithm has much fewer FLOPs than that of the other methods which have been reported. Although RL-based and GD-based architecture algorithms display similar (or slightly better) classification accuracy to that of the proposed method, our algorithm achieves preferable speed-accuracy trade-offs.

### Designed CNN architectures

**Figure 7:**
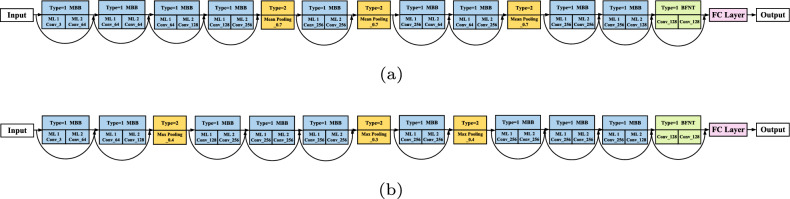
The discovered optimal CNN architectures on CIFAR10 (**a**) and CIFAR100 (**b**).

As seen from Fig. 7a, the best architecture on CIFAR10 dataset designed by proposed algorithm consists of nine multi-branch blocks, three pooling blocks and one BFNTBlock, namely containing 18 blended layers. The best architecture on CIFAR100 displayed in Fig. 7b is similar to that on CIFAR10, which also includes 18 hybrid layers. Compared with the most state-of-the-art CNNs that are solely built on basic blocks like residual block and DenseNet block, the architectures based on multi-branch blocks and BFNTBlocks automatically discovered by proposed algorithm have highly competitive performance with the less number of parameters. This means ensemble blocks may be more effective.

CIFAR100 is a more complicated benchmark dataset than CIFAR10, and CNN architectures on CIFAR100 dataset typically contain more layers than that of CIFAR10. However, according to the optimal architectures displayed in Fig. 7, the complexity of best architecture on CIFAR100 is the same as that of on CIFAR10, which indicates the proposed algorithm can design architectures with the appropriate depth according to diverse tasks.

### Evolutionary trajectory

**Figure 8:**
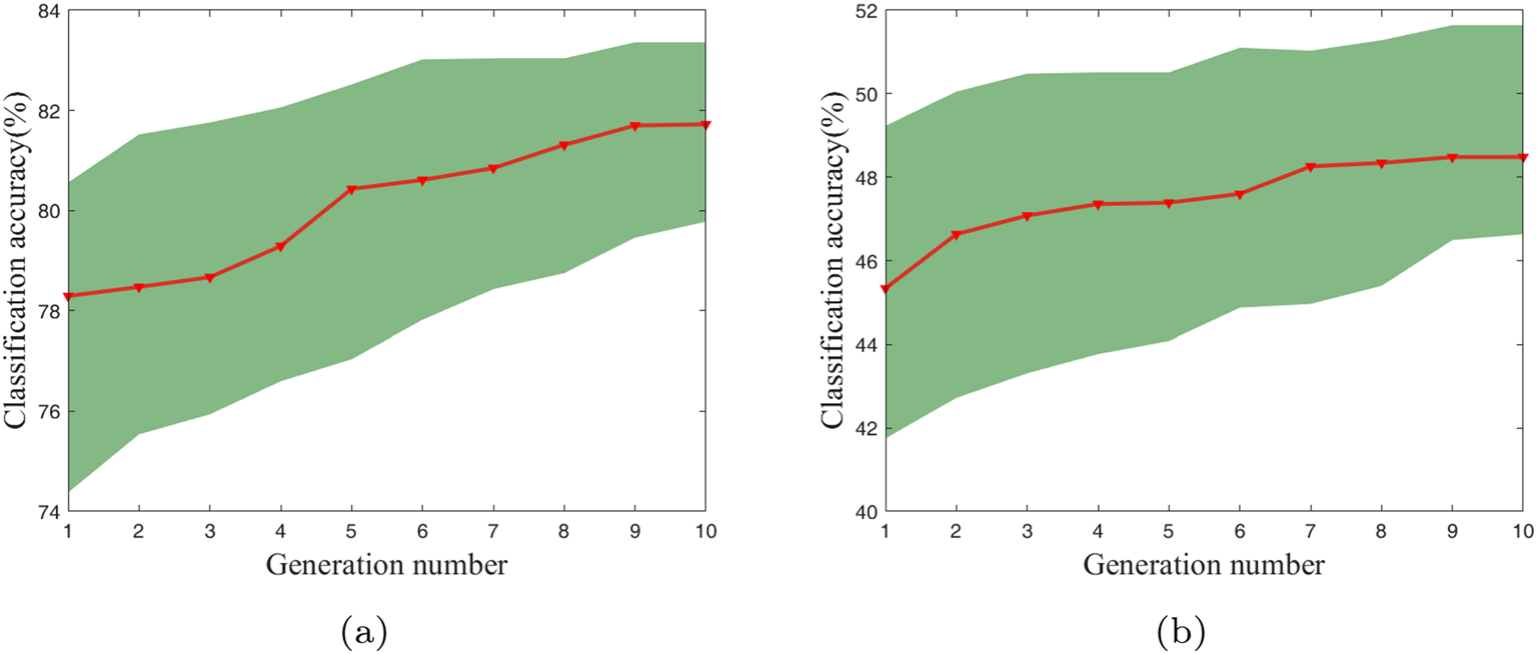
Evolutionary trajectories of the proposed algorithm on CIFAR10 (**a**) and CIFAR100 (**b**).

Aiming at intuitively illustrating the evolutionary process of discovered CNN architectures, the evolutionary trajectories of our algorithm on benchmark datasets are displayed in Fig. 8. The horizontal and vertical axis indicate generation number and classification accuracy respectively. The red line refers to the average classification accuracy of each generation, while the green area is depicted by the best and worst individual in each generation.

As depicted in Fig. 8a, the mean classification accuracy remains almost unchanged from the first generation to third generation. Due to the random initialization of population, which lead to generate inferior individuals, the mean accuracy slightly downturn at the fourth generation. Then, the individuals with uncompetitive fitness are eliminated, the mean accuracy improves and steadily moves forward until the 7th generation. After that, mean classification accuracy increases to nearly $82\%$ as the evolution process proceeds. Finally, the mean classification keep a steady state which implies the population converges. An analogous situation can be seen from Fig. 8b, the mean classification accuracy remains constant when the evolution terminates. Meanwhile, the upper and lower boundaries of the light-green areas (i.e. the best and worst classification accuracy) gradually rise and converges to a stead state. This indicates the parameter setup is reasonable since the proposed algorithm well converges. In the case of more computational resources, the evolutional generation and population size can be set to larger numbers.

### Application to traffic sign recognition task

To further demonstrate the superior performance and generalization of the proposed algorithm in real applications, we conducted experiments on GTSRB. The German Traffic Sign Recognition Benchmark (GTSRB) dataset^87^ is the standard benchmark dataset for traffic sign classification task, which contains 43 classes of traffic signs and has a total of 51,839 images, including 39,209 training images and 12,630 test images. The champion on GTSRB is MDCNN proposed by IDSIA team^88^. The MDCNN is comprised of 25 deep neural networks (DNNs) with same architecture, achieving an accuracy of $99.46\%$ on recognition accuracy.

**Figure 9:**
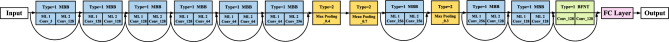
The best architecture found by proposed algorithm on GTSRB.

The optimal CNN architecture on GTSRB mainly consists of nine multi-branch blocks, three pooling blocks and one BFNTBlock (see Fig. 9). Compared with the state-of-the-art CNNs^6^, the optimal architecture comprises fewer layers and a simpler structure. In particular, the merits of discovered CNN architectures are in three folds: (1) multi-branch block possesses different paths with the ability to sufficiently extract abundant features while maintain highly competitiveness in terms of performance. (2) The proposed BFNTBlock concentrates on the aggregation of global information, which is complementary to CNNs that focus on local information. The effective aggregation between global and local information is of great significance to generate excellent network architecture. (3) The design of BN and GN mixed in the BFNTBlock obstruct the accumulated estimation shift due to the stack of BN. Table 2 refers to the classification accuracy on GTSRB obtained by state-of-the-art approaches. The proposed algorithm achieves an accuracy of 99.61$\%$ with a small number of parameters, which outperforms the human performance and other methods. This domenstrates the proposed algorithm can design network architecture efficiently and automatically for specific tasks.

**Table 2:** Comparison of the proposed algorithm with peer competitors on GTSRB dataset.

Team	Methods	Acc ($\%$)
**Proposed**	**ENAS**	**99.61**
IDSIA	Committee of CNNs	99.46
INI-RTCV	Human performance	98.84
Sermanet	Multi-scale CNNs	98.31
CAOR	Random forests on HOG	96.14
INI-RTCV	LDA on HOG	95.68

The best records are marked in bold.

### Application to defect classification

NEU-CLS is the surface defect classification dataset, containing six types of defects in the hot-rolled steel strip, each of which includes 300 images with resolution of 200 × 200. The training, validate and testing dataset are divided with the proportion of 6.4:1.6:2^89^.

The comparison results on NEU-CLS are presented in Table 3. AECLBP^90^ and BYEC^91^ are manual feature extraction methods. The Decaf^92^, ResNet34-MFN^93^ and SDC-SN-ELF+MRF^94^, are the CNN-based methods. The NAS-SDC-B^95^ is a NAS-based method. As shown in Table 3, the proposed method achieved 99.44 $\%$ prediction accuracy. It outperforms other classical methods and obtains the competitive performance against SDC-SN-ELF+MRF and NAS-SDC-B.

**Table 3:** Comparison of the proposed algorithm with peer competitors on NEU-CLS dataset.

Methods	Acc ($\%$)
AECLBP	98.93
BYEC	96.30
Decaf	99.27
ResNet34-MFN	99.17
SDC-SN-ELF+MRF	100
NAS-SDC-B	99.63
**Proposed**	**99.44**

The best records are marked in bold.

### Comparisons with state-of-the-art attention modules

To demonstrate the superiority of the BFNTBlock, we introduced different competitive attention modules in the individuals searched on three datasets to compare performance, including SE^44^, CBAM^45^, Shuffle attention^96^, Triplet attention^97^, MHSA^48^, BoTNet^53^. Evaluation criteria include the parameters of the network, accuracy and GPU days. For a fair comparison, all experiments are performed on training settings described in Sect. "Parameters settings". The baseline is MBB.

Concretely, CBAM, a lightweight and general module, infers attention maps along channel and spatial dimensions, after which are aggregated to form the feature map. Similar to CBAM, SE is also a light attention module, proposing a method to enhance the ability of the representation for the network by modelling channel-wise relationships. MHSA allows a model to jointly process information from diverse subspaces. Shuffle Attention proposes a lightweight attention module, which constructs channel attention and spatial attention simultaneously for each sub-feature. Triplet attention captures cross-dimensional interactions through a three-branch structure to calculate attention weights.

**Table 4:** Comparisons of diverse attention modules on CIFAR10, CIFAR100 and GTSRB in terms of accuracy ($\%$) and parameters(M).

Attention module	CIFAR10	CIFAR100	GTSRB
Baseline	95.14	–	–
Baseline	–	75.12	–
Baseline	–	–	98.42
SE	95.14	–	–
SE	–	77.52	–
SE	–	–	99.43
CBAM	94.92	–	–
CBAM	–	75.56	–
CBAM	–	–	98.92
MHSA	94.12	–	–
MHSA	–	75.38	–
MHSA	–	–	98.49
SA	94.94	–	–
SA	–	75.56	–
SA	–	–	99.01
TA	95.12	–	–
TA	–	75.17	–
TA	–	–	98.74
BoTNet	95.82	–	–
BoTNet	–	77.37	–
BoTNet	–	–	98.86
BFNTBlock$_{GN}$-P1	96.06	–	–
BFNTBlock$_{GN}$-P3	–	77.82	–
BFNTBlock$_{GN}$-P1	–	–	99.29

SA and TA denote Shuffle attention and Triplet attention respectively.

Table 4 represents the comparisons of the proposed BFNTBlock with diverse attention modules. It can be observed that the BFNTBlock enhances classification performance by $0.92\%$, $2.7\%$, $0.87\%$ on CIFAR10, CIFAR100 and GTSRB respectively compared with the baseline. Note that the classification accuracy on CIFAR10 degrades after incorporating CBAM, SA and MHSA due to the lack of adaptability. The experimental results verify that the proposed BFNTBlock can be better integrated with architectures designed by the proposed algorithm, and achieves the optimal performance improvement. The improvements mainly benefit from the followings: (1) the proposed BFNTBlock leverages the merits of both MHSA and convolutions, including global receptive field and inductive bias (2) GN employed in the BFNTBlock obstruct the accumulative estimation shift arising from the stack of BN.

### Advanced training strategies

Besides the standard training strategies described in Sect. "Parameters settings", we adopt advanced training strategies to augment the training data: (1) cutout^98^ with a patch length of 16, 8, 2 on CIFAR10, CIFAR100 and GTSRB respectively (2) mixup^99^ training with a mix factor of 0.2. ‘ST’ indicates standard training. The baseline model comprises MBB and BoTNet. As shown in Table 5, the networks with BFNTBlock generated by proposed algorithm also consistently outperforms the baseline by a remarkable margin. This reconfirms the remarkable performance of the proposed BFNTBlock.

**Table 5:** The accuracy ($\%$) on CIFAR10, CIFAR100 and GTSRB using advanced training strategies.

Training strategies	CIFAR10		CIFAR100		GTSRB	
	Baseline	$\hbox {BFNTBlock}_{GN}$-P1	Baseline	$\hbox {BFNTBlock}_{GN}$-P3	Baseline	$\hbox {BFNTBlock}_{GN}$-P2
ST	95.82	96.06	77.37	77.82	98.86	98.95
MixUp	94.62	96.50	76.67	78.62	99.33	99.42
CutOut	95.68	96.80	75.84	79.17	99.51	99.43
MixUp+CutOut	96.04	97.24	77.92	80.86	99.53	99.61

### Ablation studies

#### Positions of BFN in a BFNTBlock

We investigate the position of BFN applied in the BFNTBlock. For fair comparison, all experiments are conducted on the settings according to Sect. "Parameters settings". We use GN (group number=64) as BFN in this paper. We design three BFNTBlock variants, which replace the first, second and third BN in the BoTNet (referred as ’BFNTBlock$_{GN}$-P1’, ’BFNTBlock$_{GN}$-P2’ and ’BFNTBlock$_{GN}$-P3’). We substitute these BFNTBlocks for BoTNet and report the results in Table 6. Our baseline is the model containing MBB and BoTNet. Table 6 shows the comparison results of applying a GN at different positions in the BFNTBlock. It can be discovered that in addition to the slight performance decline of BFNTBlock$_{GN}$-P3 on CIFAR10 and that of BFNTBlock$_{GN}$-P1 on CIFAR100, the performances of other networks with BFNTBlock outperform the baseline to an extent. This further verify the comprehensive availability of our BFNTBlock.

**Table 6:** Results of GN applied at the different positions in a BFNTBlock.

Methods	CIFAR10	CIFAR100	GTSRB
Baseline	95.82	77.37	98.86
$\hbox {MBB+BFNTBlock}_{GN}$-P1	96.06${\textbf {(+0.24)}}$	77.31${\textbf {(-0.06)}}$	99.29${\textbf {(+0.43)}}$
$\hbox {MBB+BFNTBlock}_{GN}$-P2	95.90${\textbf {(+0.08)}}$	77.43${\textbf {(+0.06)}}$	98.95${\textbf {(+0.09)}}$
$\hbox {MBB+BFNTBlock}_{GN}$-P3	95.76${\textbf {(-0.06)}}$	77.82${\textbf {(+0.45)}}$	98.94${\textbf {(+0.08)}}$

We evaluate the test classification accuracy ($\%$) on CIFAR10, CIFAR100 and GTSRB datasets. Bold indicates the corresponding improvement ratio.

#### Comprehensive analysis for components of proposed algorithm

We thoroughly analyse the components of the proposed algorithm and validate the contribution of the designed BFNTBlock to improve the expression ability of CNN architecture. First, we conduct experiments on the baseline model merely containing multi-branch block. After that, considering the MHSA is one of the most essential components of BoTNet, on which the proposed BFNTBlock is designed, the MHSA, BoTNet and BFNTBlock are sequentially incorporated into neural architectures to test performance. All parameters are consistent with configurations set in Sect. "Parameters settings".

It can be obviously observed from Table 7, the performance on CIFAR10 remarkably descends when only incorporate MHSA due to the lack of normalization, which has a great impact on learning robust significant coefficients and may lead to degradation of performance. However, the performance on CIFAR10, CIFAR100 and GTSRB remarkably improve when the proposed BFNTBlock is introduced in the discovered architecture. BFNTBlock sufficiently leverages the advantages of convolutions and MHSA. Convolutional layer possesses better generalization ability with faster convergence due to its inherent prior of inductive bias, while MHSA layer has larger model capacity that can benefit from larger datasets. In particular, BFNTBlock obstruct accumulation of estimation shift, which ensures a significant performance improvement. Furthermore, BFNTBlock is a lightweight and powerful module, it can be seamlessly integrated into CNN architectures with negligible overheads. The experimental results demonstrate all components in our algorithm are indispensable for the improvement of performance.

**Table 7:** Performance comparisons of the proposed algorithm with different components.

Methods	CIFAR10	CIFAR100	GTSRB	Parameters
MBB	95.14	-	-	3.81
MBB	–	75.12	–	4.08
MBB	–	–	98.42	1.62
MBB + MHSA	94.12	–	–	3.9
MBB + MHSA	–	75.38	–	2.92
MBB + MHSA	–	–	98.49	3.73
MBB + BoTNet	95.82	–	–	4.73
MBB + BoTNet	–	77.37	–	5.71
MBB + BoTNet	–	–	98.86	3.52
MBB + BFNTBlock$_{GN}$-P1	96.06	–	–	4.73
MBB + BFNTBlock$_{GN}$-P3	–	77.82	–	5.71
MBB + BFNTBlock$_{GN}$-P1	–	–	99.29	3.52

## Conclusions and future work

This article proposed an efficient ENAS algorithm combing multi-branch ConvNet with batch-free normalization Transformer backbone to evolve compact CNN architectures for image classification task. To effectively search network architectures with promising performance, a search space was designed based on multi-branch block and the proposed BFNTBlock. Furthermore, a flexible encoding strategy was developed to adaptively evolve the configurations of CNN in a variable-length manner. The designed crossover and mutation operators provide effective local search and global search ability for our proposed algorithm. The computational complexity of the algorithm is significantly reduced by a parallel computational component. The proposed algorithm is examined on two challenging classification benchmark datasets, CIFAR10 and CIFAR100, achieving accuracies of $97.24\%$, $80.06\%$, respectively, with low search cost. The proposed algorithm is superior to almost all the hand-crafted architectures and ENAS algorithms, and achieves a competitive performance among the non-EAs-based NAS competitors. Furthermore, the proposed algorithm obtains competitive performance on two real-world applications, including the GTSRB and NEU-CLS dataset.

A limitation of our approach is the search space based on richly experienced building blocks limit the diversity of architectures. More flexible search spaces deserve to be explored. Extending NAS methods to other complicated downstream tasks like semantic segmentation and object detection is another meaningful future work. In addition, we plan to focus on developing effective evolutionary methods to notably accelerate the process of fitness evaluation.

## Data Availability

All data generated or analysed during the current study are available from the corresponding author on reasonable request.
